# Anthropometric and physiologic characteristics in white and British Indian vegetarians and nonvegetarians in the UK Biobank

**DOI:** 10.1093/ajcn/nqy042

**Published:** 2018-06-04

**Authors:** Tammy YN Tong, Timothy J Key, Jakub G Sobiecki, Kathryn E Bradbury

**Affiliations:** 1 Cancer Epidemiology Unit, Nuffield Department of Population Health, University of Oxford, Oxford, United Kingdom; 2 Department of Epidemiology and Biostatistics, School of Public Health, Imperial College London, London, United Kingdom

**Keywords:** vegetarian, diet group, anthropometry, blood pressure, bone mineral density, grip strength, pulse rate, UK Biobank

## Abstract

**Background:**

A detailed description of anthropometric and physiologic characteristics of persons in different diet groups is lacking.

**Objective:**

We aimed to perform cross-sectional analyses of diet group with anthropometric and physiologic characteristics in a large cohort in the United Kingdom.

**Design:**

The UK Biobank recruited ∼500,000 middle-aged participants throughout the United Kingdom in 2006–2010. Anthropometric indexes (height, weight, waist and hip circumference, body composition) and other physiologic characteristics (heel bone mineral density, grip strength, blood pressure, pulse rate) were measured following standardized protocols. We estimated the age-adjusted means of each characteristic in 6 diet groups (198,166 regular meat eaters, 199,784 low meat eaters, 4381 poultry eaters, 9674 fish eaters, 6366 vegetarians, and 378 vegans) in white women and men, and in 2 diet groups (3322 meat eaters and 1186 vegetarians) in British Indian women and men.

**Results:**

In white women, after adjustment for age and compared with regular meat eaters, non–red meat eaters had lower adiposity (e.g., 4.5% lower body fat in vegan women) and lower systolic and diastolic blood pressure (−4.2 and −3.3 mm Hg, respectively), and generally lower heel bone mineral density t-score (−0.26). Patterns of differences by diet group were similar in white men. In the Indian population, compared with meat eaters, vegetarian women were shorter (−1.1 cm) and had lower lean mass (−0.5 kg), and both vegetarian women and men had lower grip strength (−1.3 and −1.4 kg, respectively). No significant differences in the other characteristics were observed.

**Conclusions:**

Differences in anthropometric and physiologic characteristics were observed across diet groups in white participants, but fewer differences were observed in British Indian participants. The observed differences may be important as intermediate markers of long-term health in different diet groups. This observational study was registered at http://www.isrctn.com/ as ISRCTN10125697.

## INTRODUCTION

Previous literature indicates that vegetarians generally have lower, and therefore more optimal, BMI, waist circumference, and blood pressure than meat eaters (1–3). However, less is known about differences between diet groups in other body-composition measures or physiologic characteristics such as bone mineral density (BMD), grip strength, or pulse rate (4–6). Overall, few large studies have comprehensively assessed anthropometric and physiologic characteristics by detailed categorization of diet groups.

Because differences in anthropometric measures and other physical attributes may, in turn, influence the risk of overall mortality and the incidence of noncommunicable diseases such as diabetes, cardiovascular diseases, and fractures (7–10), an understanding of the link between diet group and these characteristics is important for establishing mechanisms that link diet to disease outcomes. Therefore, our aim in this study was to present a detailed description of anthropometric indexes and physiologic measures across white and British Indian participants in different habitual diet groups with the use of data from a large-population cohort, the UK Biobank.

## METHODS

### Study design and participants

The UK Biobank study is a prospective cohort of >500,000 people aged 40–69 y, who were recruited in 2006–2010 across the United Kingdom (11). The scientific rationale and design of the UK Biobank study has been described in detail elsewhere (12). In brief, persons who lived within reasonable traveling distance (∼25 km) of 1 of the 22 assessment centers across England, Wales, and Scotland were identified from National Health Service registers and invited to participate in the study. Permission for access to patient records for recruitment was approved by the Patient Information Advisory Group (subsequently replaced by the National Information Governance Board for Health and Social Care) in England and Wales and the Community Health Index Advisory Group in Scotland. Overall, ∼5.5% of the invitees attended a baseline visit (13) during which they gave informed consent to participate in the UK Biobank using a signature capture device and completed a touch-screen questionnaire that asked about sociodemographic characteristics, lifestyle exposures (smoking, diet and alcohol intake, and physical activity), and general health and medical history. For this study, we excluded participants who reported that they had changed their diet in the past 5 y due to illness or who did not answer this question (*n* = 57,907). All of the participants also attended a computer-assisted personal interview and had physical measurements taken. In addition to the touch-screen questionnaire, additional dietary information was collected with the use of a Web-based 24-h dietary assessment tool (14), which was administered ≤5 times in a large subsample of participants (∼210,000). A participant flow chart of this study is included as **Supplemental Figure 1**. This observational study was registered at http://www.isrctn.com/ as ISRCTN10125697.

### Ethnicity classification

On the touch-screen questionnaire, participants were asked to select their ethnicity from options of “White,” “Mixed,” “Asian or Asian British,” “Black or Black British,” “Chinese,” “Other ethnic group,” “Do not know,” or “Prefer not to answer.” Participants were included for analyses if they identified as “white” or as “Asian or Asian British” and subsequently as “Indian.” The white population was included because it made up the majority of the UK Biobank population (∼94%), and the British Indian population was included due to the large proportion of vegetarians in this population group (24.6% compared with 1.7% in the overall cohort).

### Diet group classification

For classification of diet groups, relevant dietary information from the touch-screen questionnaire was used. Participants were asked their frequency of consumption of processed meat, beef, lamb or mutton, pork, chicken, turkey or other poultry, oily fish, or other types of fish, in 6 categories of frequency ranging from “Never” to “Once or more daily.” Participants were also asked whether they never ate eggs or foods containing eggs or dairy products.

On the basis of these questions, 6 diet groups (regular meat eaters, low meat eaters, poultry eaters, fish eaters, vegetarians, and vegans) were defined for the white population. To rank the participants by weekly red and processed-meat consumption based on the touch-screen, we summed the frequencies for processed meat, beef, pork, and lamb or mutton by using the following coding: “Never” = 0, “Less than once a week” = 0.5, “Once a week” = 1, “2–4 times a week” = 3, “5–6 times a week” = 5.5, and “Once or more daily” = 7. Regular meat eaters were defined as participants who reported eating red (beef, lamb or mutton, or pork) or processed meat >3 times/wk, whereas low meat eaters were defined as participants who reported eating red or processed meat ≤3 times/wk, regardless of what else they ate. Poultry eaters were defined as participants who reported never consuming any red or processed meat but who did eat poultry. Fish eaters were participants who reported that they never consumed any red meat, processed meat, or poultry but who ate oily or nonoily fish. Vegetarians were defined as participants who reported that they never consumed any meat or fish, and vegans were participants who reported that they never consumed any meat, fish, eggs or foods containing eggs, or dairy products. A total of 2630 white participants who did not answer the relevant questions to be classified into a diet group were excluded from our analyses. For the British Indian population, 2 diet groups (meat eaters and vegetarians) were defined due to the smaller numbers in this population group. Meat eaters were defined as participants who reported eating any red meat, processed meat, or poultry; and vegetarians were defined as participants who reported that they did not eat any meat or fish but did eat eggs, dairy products, or both. A total of 278 British Indian participants were excluded because they did not answer the relevant questions, or because they were fish eaters or vegans. Separately, information collected with the use of the Web-based 24-h dietary assessment tool was used to estimate food and nutrient intakes in each diet group.

### Anthropometric and physiologic measures

Anthropometric and physiologic measures were collected on all participants during the baseline visit by trained staff following a standardized protocol. At the physical measures station, participants were asked to remove their socks and shoes. Height was measured with the use of the Seca 202 height measure (Seca, Hamburg, Germany). Waist (at the natural indent) and hip (widest point) circumferences were measured over light clothes with the use of the Seca-200 tape measure (Seca, Hamburg, Germany). Weight and bioimpedance were measured by using the Tanita BC418ma bioimpedance device (Tanita, Tokyo, Japan), from which percentages of body fat and lean mass were estimated. BMI was calculated as weight (kilograms)/height (meters) squared. Calcaneal bone density was taken on the left heel with the use of a Norland McCue Contact Ultrasound Bone Analyzer (Norland, Trumbull, Connecticut, USA), with participants sitting upright; and a heel BMD t-score was calculated on the basis of the measured BMD values. Hand-grip strength for each hand was taken by using the Jamar Hydraulic hand dynamometer (Lafayette Instrument Company, Lafayette, Indiana, USA), and we considered both the higher grip-strength value of either hand and average grip strength of the 2 hands for our analyses. Systolic blood pressure (SBP), diastolic blood pressure (DBP), and pulse rate were taken with the use of the Omron HEM-7015IT digital blood pressure monitor (Omron, Kyoto, Japan) after participants have been seated for ≥5 min, and the averages of 2 measurements, taken ≥1 min apart, were used for analyses.

### Statistical analyses

Summary baseline characteristics of the cohort were tabulated by 6 diet groups in white women and men and by 2 diet groups in British Indian women and men. With the use of linear regression, we estimated the age-adjusted (5-y age groups from <45, 45–49, 50–54, 55–59, 60–64, and ≥65 y) means of the anthropometric and physiologic characteristics in each diet group. The characteristics studied were height, weight, BMI, waist circumference, hip circumference, body fat percentage, lean mass, heel BMD (as original values and as a t-score), grip strength (as original values and as per kilogram of lean mass), SBP, DBP, and pulse rate, all modeled continuously. Subsequently, we additionally adjusted for body weight (as 2.5-kg categories) in the analyses for heel BMD t-score; height (as 2.5-cm categories), lean mass (as 2.5-kg categories), and physical activity (as 5-unit categories in excess metabolic equivalent-hours per week) in the analyses for grip strength; and body fat percentage (as 2.5% categories) in the analyses for SBP, DBP, and pulse rate. For all covariates, missing values were coded as a missing category in the adjustment. For each baseline characteristic and each association, post hoc pairwise comparisons based on linear regression models were used to test for significant differences between the diet groups in both white and British Indian populations, with Bonferroni correction for multiple comparisons in the white population. All statistical analyses were performed with the use of Stata release 14.1 (StataCorp, College Station, Texas, USA), and 2-sided *P* values <0.05 were considered significant.

## RESULTS

### Baseline characteristics

Together, 229,806 white women (86,432 high meat eaters, 128,429 low meat eaters, 3429 poultry eaters, 6988 fish eaters, 4305 vegetarians, and 223 vegans), 188,943 white men (111,734 regular meat eaters, 71,355 low meat eaters, 952 poultry eaters, 2686 fish eaters, 2061 vegetarians, and 155 vegans), 2183 Indian women (1422 meat eaters and 761 vegetarians), and 2325 Indian men (1900 meat eaters and 425 vegetarians) were included in our analyses. Overall, 98% of the non-meat eaters (fish eaters, vegetarians, and vegans) had not eaten any meat for ≥1 y, and 92% (89% in fish eaters to 96% in vegetarians) had not eaten any meat for ≥5 y.

In the white population, fish eaters, vegetarians, and vegans were generally younger, of lower area-level socioeconomic status [measured by the Townsend score (15)], more educated, and less likely to smoke than were regular and low meat eaters and poultry eaters (**Tables 1** and **2**). Fish eaters and vegetarians were less likely to report long-standing illness. In white women, vegetarians were more likely to have active jobs, but the opposite was observed in white men. Overall, vegans had the highest percentage of energy from carbohydrates but the lowest percentage of energy from protein, total fat, and saturated fat.

**TABLE 1:** Baseline characteristics of white women by diet group in the UK Biobank^1^

	Meat eaters^2^					
Characteristics	Regular consumption (>3 times/wk) (max *n* = 86,432)	Low consumption (≤3 times/wk) (max *n* = 128,429)	Poultry eaters (max *n* = 3429)	Fish eaters (max *n* = 6988)	Vegetarians (max *n* = 4305)	Vegans (max *n* = 223)
Age, y	56.6 ± 8.0^c^	56.5 ± 7.9^c^	56.5 ± 8.0^c^	54.0 ± 8.0^a^	52.8 ± 7.8^b^	54.4 ± 8.0^a^
Top socioeconomic quintile,^3^*n* (%)	18,784 (21.8)^d^	27,337 (21.3)^c,d^	673 (19.6)^b,c^	1259 (18.0)^a,b^	701 (16.3)^a^	26 (11.7)^a,b^
Has a degree or vocational qualification, *n* (%)	46,736 (54.9)^d^	73,052 (57.8)^c^	2099 (62.4)^b^	5227 (75.8)^a^	3192 (74.9)^a^	167 (75.6)^a^
Smoking status, *n* (%)						
Previous	26,965 (31.3)	41,843 (32.7)	1184 (34.7)	2572 (36.9)	1380 (32.1)	81 (36.3)
Current	8549 (9.9)^c^	10,709 (8.4)^b^	261 (7.6)^a,b,c^	463 (6.6)^b,c^	295 (6.9)^a^	15 (6.7)^a,b,c^
Has a long-standing illness, *n* (%)	22,841 (27.1)^b^	30,708 (24.5)^a^	835 (24.8)^a^	1605 (23.4)^a^	1016 (24.2)^a^	61 (27.9)^a,b^
Physical activity, *n* (%)						
Moderate	33,703 (53.6)	53,280 (54.9)	1469 (54.5)	3280 (58.0)	1940 (55.3)	89 (49.2)
High	13,002 (20.7)^d^	20,789 (21.4)^a^	777 (28.8)^c^	1308 (23.1)^b^	830 (23.6)^b^	55 (30.4)^a,b,c^
Has an active job, *n* (%)	15,692 (18.2)^a^	24,085 (18.8)^b^	664 (19.4)^a,b,c^	1341 (19.2)^a,b,c^	911 (21.2)^c^	52 (23.3)^a,b,c^
Intake						
Alcohol, g/d	12.4 ± 12.1^c^	10.8 ± 10.5^a,b^	10.3 ± 11.0^a^	11.2 ± 11.2^b^	10.6 ± 11.5^a,b^	8.6 ± 8.1^a,b^
Total fruit and vegetables, servings/d	5.0 ± 2.6^d^	5.5 ± 2.7^c^	6.4 ± 3.4^b^	6.4 ± 3.3^b^	6.3 ± 3.4^b^	8.0 ± 6.4^a^
Red and processed meat,^4^ g/d	66.4 ± 57.2^d^	46.6 ± 50.6^c^	9.2 ± 27.4^b^	2.0 ± 14.0^a^	0.6 ± 8.5^a^	1.2 ± 9.3^a,b^
Poultry,^4^ g/d	32.1 ± 48.2^b^	32.3 ± 48.3^b^	30.9 ± 47.6^b^	1.3 ± 10.4^a^	0.1 ± 2.5^a^	1.2 ± 11.6^a^
Nonoily fish,^4^ g/d	14.8 ± 33.2^d^	15.6 ± 33.7^b^	18.0 ± 33.3^b,c^	18.5 ± 35.2^c^	0.6 ± 6.3^a^	0.4 ± 3.5^a^
Oily fish,^4^ g/d	10.1 ± 24.8^d^	12.2 ± 27.1^c^	18.0 ± 33.6^b^	17.6 ± 32.4^b^	0.4 ± 4.2^a^	0.4 ± 4.3^a^
Total energy,^4^ kJ/d	8360 ± 2102^d^	7915 ± 2033^b^	7702 ± 2169^a^	8026 ± 2043^c^	7959 ± 2173^b,c^	7790 ± 2281^a,b,c^
Energy from carbohydrates, %	45.2 ± 7.7^e^	46.6 ± 7.8^d^	48.2 ± 8.6^c^	48.8 ± 7.9^c^	50.4 ± 7.8^b^	53.9 ± 8.0^a^
Energy from protein, %	16.9 ± 3.7^e^	16.7 ± 3.8^d^	16.0 ± 3.8^c^	14.3 ± 2.9^b^	13.2 ± 2.6^a^	12.6 ± 2.5^a^
Energy from fat, %	33.4 ± 6.6^c^	32.2 ± 6.8^b^	31.7 ± 7.5^a^	32.5 ± 7.1^b^	32.5 ± 7.1^b^	30.0 ± 7.4^a^
Energy from saturated fat, %	12.8 ± 3.3^e^	12.2 ± 3.3^c^	11.6 ± 3.6^d^	11.9 ± 3.4^b,d^	12.1 ± 3.6^b,c^	8.4 ± 3.1^a^

^1^Values are means ± SDs unless otherwise indicated; *n* = 229,806. Groups that do not share a superscript letter were significantly different at the 5% level from post hoc pairwise comparisons based on linear regression models and after Bonferroni correction for multiple comparisons. For categorical variables, this referred to overall differences across strata. max, maximum.

^2^Includes participants who consume any red or processed meat, regardless of whether they consume poultry, fish, or dairy. Cutoffs of regular and low consumption were determined on the basis of consumption of red and processed meat (beef, lamb, pork, processed meat) as reported on the touch-screen questionnaire.

^3^The least-deprived quintile based on the Townsend deprivation index.

^4^Based on 100,282 white women who completed ≥1 Web-based 24-h dietary assessment and after exclusion of implausible energy intakes (>18,000 kJ for women) and participants who reported any consumption of other hot or cold beverages (UK Biobank variable data field ID 100560), due to inaccurate energy coding for this variable. The max numbers for these variables in white women were as follows: 36,248 meat eaters of regular consumption, 56,141 meat eaters of low consumption, 1523 poultry eaters, 3779 fish eaters, 2420 vegetarians, and 123 vegans.

**TABLE 2:** Baseline characteristics of white men by diet group in the UK Biobank^1^

	Meat eaters^2^					
Characteristics	Regular consumption (>3 times/wk) (max *n* = 111,734)	Low consumption (≤3 times/wk) (max *n* = 71,355)	Poultry eaters (max *n* = 952)	Fish eaters (max *n* = 2686)	Vegetarians (max *n* = 2061)	Vegans (max *n* = 155)
Age, y	56.7 ± 8.2^c^	57.0 ± 8.1^d^	56.7 ± 8.3^c,d^	54.3 ± 8.0^b^	52.6 ± 7.9^a^	53.8 ± 7.7^a,b^
Top socioeconomic quintile,^3^*n* (%)	23,760 (21.3)^b^	16,042 (22.5)^c^	154 (16.2)^a^	417 (15.5)^a^	312 (15.2)^a^	21 (13.5)^a,b,c^
Has a degree or vocational qualification, *n* (%)	69,971 (63.6)^a^	46,559 (66.3)^b^	637 (68.1)^a,b^	2103 (79.1)^c^	1585 (77.6)^c^	109 (71.2)^a,b,c^
Smoking status, *n* (%)						
Previous	42,939 (38.5)	27,311 (38.4)	328 (34.5)	1013 (37.8)	734 (35.7)	71 (46.1)
Current	15,584 (14.0)^c^	6845 (9.6)^b^	68 (7.2)^a^	227 (8.5)^a,b^	201 (9.8)^a,b^	13 (8.4)^a,b,c^
Has a long-standing illness, *n* (%)	34,471 (31.5)^c^	20,004 (28.6)^b^	262 (28.0)^a,b,c^	669 (25.4)^a^	551 (27.2)^a,b^	55 (36.4)^a,b,c^
Physical activity, *n* (%)						
Moderate	46,201 (50.3)	31,361 (52.9)	403 (50.3)	1319 (57.0)	963 (53.9)	87 (62.6)
High	22,929 (25.0)^a^	14,501 (24.5)^b^	272 (34.0)^d^	606 (26.2)^c^	438 (24.5)^a,b,c^	33 (23.7)^a,b,c,d^
Has an active job, *n* (%)	25,801 (23.1)^b^	14,742 (20.7)^a,b^	194 (20.4)^a,b^	491 (18.3)^a^	419 (20.3)^a^	28 (18.1)^a,b^
Intake						
Alcohol, g/d	27.4 ± 25.0^c^	21.4 ± 19.6^b^	17.6 ± 16.1^a^	20.7 ± 18.5^b^	20.2 ± 21.8^a,b^	16.8 ± 19.2^a,b^
Total fruit and vegetables, servings/d	4.4 ± 2.6^e^	5.0 ± 2.9^d^	6.3 ± 3.7^c^	6.0 ± 3.3^b,c^	5.9 ± 3.3^b^	8.4 ± 6.3^a^
Red and processed meat,^4^ g/d	80.1 ± 68.4^c^	55.7 ± 58.0^b^	8.7 ± 27.7^a^	1.7 ± 11.9^a^	0.9 ± 11.0^a^	0.0 ± 0.0^a^
Poultry,^4^ g/d	31.5 ± 51.3^b^	32.0 ± 50.5^b^	34.6 ± 53.1^b^	0.9 ± 9.7^a^	0.4 ± 6.6^a^	0.0 ± 0.0^a^
Nonoily fish,^4^ g/d	16.1 ± 38.6^c^	16.4 ± 36.8^c^	22.7 ± 43.6^b^	21.9 ± 40.8^b^	0.7 ± 7.9^a^	0.0 ± 0.0^a^
Oily fish,^4^ g/d	9.7 ± 26.5^e^	12.6 ± 29.1^d^	26.3 ± 48.7^c^	18.6 ± 37.4^b^	0.6 ± 6.4^a^	0.0 ± 0.0^a^
Total energy,^4^ kJ/d	9690 ± 2509^c^	9101 ± 2379^a^	9129 ± 2642^a,b^	9433 ± 2403^b^	9460 ± 2551^b^	8961 ± 2655^a,b,c^
Energy from carbohydrates, %	44.3 ± 7.9^e^	46.3 ± 7.9^d^	49.3 ± 8.6^b,c^	48.6 ± 7.9^c^	49.5 ± 7.8^b^	56.3 ± 7.7^a^
Energy from protein, %	15.8 ± 3.5^c^	15.8 ± 3.5^c^	15.5 ± 3.5^c^	13.9 ± 2.8^b^	12.7 ± 2.3^a^	12.5 ± 2.5^a^
Energy from fat, %	32.7 ± 6.6^c^	31.4 ± 6.7^d^	30.3 ± 7.4^e^	31.8 ± 6.8^b,d^	32.6 ± 7.2^b,c^	28.0 ± 7.4^a^
Energy from saturated fat, %	12.6 ± 3.3^e^	12.0 ± 3.3^b^	10.6 ± 3.7^d^	11.5 ± 3.4^c^	12.0 ± 3.6^b^	7.6 ± 3.0^a^

^1^Values are means ± SDs unless otherwise indicated; *n* = 188,943. Groups that do not share a superscript letter were significantly different at the 5% level from post hoc pairwise comparisons based on linear regression models and after Bonferroni correction for multiple comparisons. For categorical variables, this referred to overall differences across strata. max, maximum.

^2^Includes participants who consume any red or processed meat, regardless of whether they consume poultry, fish, or dairy. Cutoffs of regular and low consumption were determined on the basis of consumption of red and processed meat (beef, lamb, pork, processed meat) as reported on the touch-screen questionnaire.

^3^The least-deprived quintile based on the Townsend deprivation index.

^4^Based on 80,585 white men who completed ≥1 Web-based 24-h dietary assessment and after exclusion of implausible energy intakes (>20,000 kJ for men) and participants who reported any consumption of other hot or cold beverages (UK Biobank variable data field ID 100560), due to inaccurate energy coding for this variable. The max numbers for these variables in white men were as follows: 46,093 meat eaters of regular consumption, 31,516 meat eaters of low consumption, 439 poultry eaters, 1435 fish eaters, 1149 vegetarians, and 86 vegans.

In the British Indian population, vegetarians were slightly older and less likely to smoke than were meat eaters (**Table 3**). Overall, British Indian vegetarians also had lower alcohol consumption, a higher percentage of energy from carbohydrates, and a lower percentage of energy from protein and fat than Indian meat eaters.

**TABLE 3:** Baseline characteristics of British Indian women and men by diet group in the UK Biobank^1^

	Women		Men	
Characteristics	Meat eaters (max *n* = 1422)	Vegetarians (max *n* = 761)	Meat eaters (max *n* = 1900)	Vegetarians (max *n* = 425)
Age, y	52.5 ± 8.1^b^	54.1 ± 7.8^a^	53.8 ± 8.6^b^	55.5 ± 8.6^a^
Top socioeconomic quintile,^2^*n* (%)	161 (11.3)^a^	75 (9.9)^a^	202 (10.6)^a^	49 (11.5)^a^
Has a degree or vocational qualification, *n* (%)	841 (61.8)^b^	385 (54.7)^a^	1197 (65.5)^a^	255 (62.0)^a^
Smoking status, *n* (%)				
Previous	89 (6.3)	12 (1.6)	355 (18.9)	60 (14.3)
Current	60 (4.2)^b^	3 (0.4)^a^	245 (13.0)^b^	22 (5.3)^a^
Has a long-standing illness, *n* (%)	300 (21.9)^a^	144 (19.8)^a^	427 (23.4)^a^	102 (24.8)^a^
Physical activity, *n* (%)				
Moderate	501 (51.0)	245 (50.5)	744 (51.4)	163 (51.4)
High	178 (18.1)^a^	75 (15.5)^a^	299 (20.7)^b^	42 (13.2)^a^
Has an active job, *n* (%)	355 (25.2)^a^	203 (26.9)^a^	589 (31.2)^a^	116 (27.6)^a^
Intake				
Alcohol, g/d	5.7 ± 8.3^b^	3.0 ± 4.9^a^	17.7 ± 19.4^b^	7.0 ± 11.4^a^
Total fruit and vegetables, servings/d	6.1 ± 4.0^b^	7.4 ± 4.4^a^	6.1 ± 4.6^b^	7.6 ± 6.0^a^
Red and processed meat,^3^ g/d	41.0 ± 65.9^b^	0.6 ± 9.3^a^	46.7 ± 67.5^b^	0.0 ± 0.0^a^
Poultry,^3^ g/d	34.9 ± 60.4^b^	0.2 ± 3.3^a^	41.6 ± 74.2^b^	0.2 ± 2.7^a^
Nonoily fish,^3^ g/d	13.7 ± 36.1^b^	0.0 ± 0.0^a^	15.1 ± 40.1^b^	0.3 ± 3.9^a^
Oily fish,^3^ g/d	10.8 ± 31.6^b^	0.2 ± 3.4^a^	7.0 ± 29.5^b^	0.0 ± 0.0^a^
Total energy,^3^ kJ/d	7535 ± 2622^a^	7114 ± 2789^a^	8490 ± 3005^b^	7466 ± 2935^a^
Energy from carbohydrates,%	49.6 ± 9.0^b^	57.4 ± 7.9^a^	49.5 ± 10.0^b^	58.1 ± 8.4^a^
Energy from protein, %	16.4 ± 4.7^b^	12.7 ± 2.3^a^	15.8 ± 4.3^b^	12.8 ± 2.6^a^
Energy from fat, %	32.0 ± 6.8^b^	29.2 ± 7.5^a^	30.2 ± 7.5^b^	28.1 ± 7.2^a^
Energy from saturated fat, %	11.4 ± 3.2^b^	10.3 ± 3.6^a^	10.7 ± 3.4^a^	10.3 ± 3.7^a^

^1^Values are means ± SDs unless otherwise indicated; *n* women = 2183, *n* men = 2325. Groups that do not share a superscript letter were significantly different at the 5% level from post hoc pairwise comparisons based on linear regression models. Comparisons were made separately for women and men. max, maximum.

^2^The least-deprived quintile based on the Townsend deprivation index.

^3^Based on 734 Indian women and 792 Indian men who completed ≥1 Web-based 24-h dietary assessment and after exclusion of implausible energy intakes (>18,000 kJ for women and >20,000 kJ for men) and participants who reported any consumption of other hot or cold beverages (UK Biobank variable data field ID 100560), due to inaccurate energy coding for this variable. The max numbers for these variables in Indian women and men were as follows: 515 meat eaters and 219 vegetarians among women and 648 meat eaters and 144 vegetarians among men.

### Anthropometric and physiologic characteristics

**FIGURE 1:**
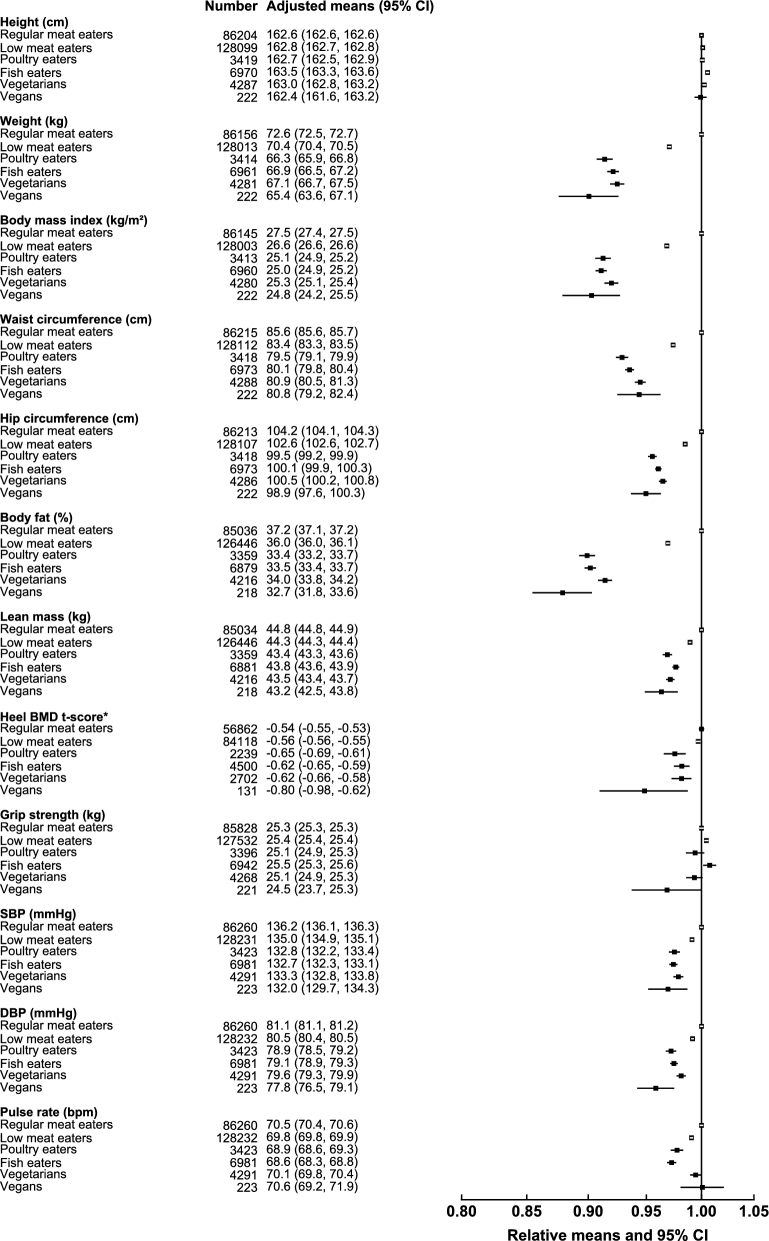
Anthropometric indexes and physiologic characteristics of white women by diet group in the UK Biobank. Regular meat eaters and low meat eaters were defined on the basis of consumption of red and processed meat >3 times or ≤3 times/wk. All characteristics are presented as age-adjusted means (5-y age groups) and as relative means, with regular meat eaters as the reference category. Estimates are modeled on the basis of linear regression. *For heel BMD, adjusted means were calculated on the basis of t-score, but relative means were plotted on the basis of the original values for interpretability. BMD, bone mineral density; bpm, beats per minute; DBP, diastolic blood pressure; SBP, systolic blood pressure.

**FIGURE 2:**
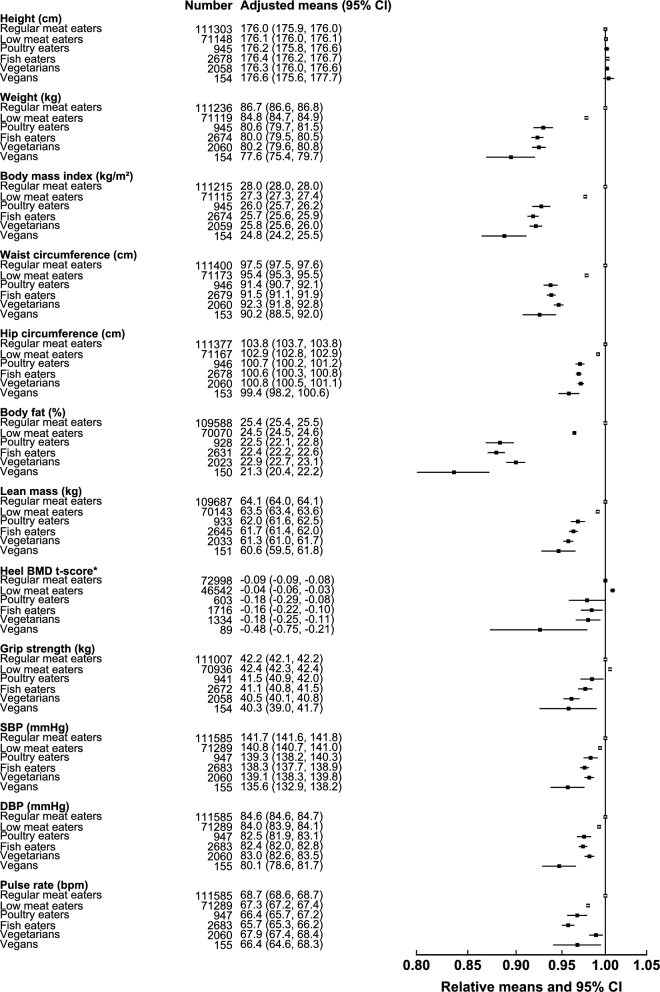
Anthropometric indexes and physiologic characteristics of white men by diet group in the UK Biobank. Regular meat eaters and low meat eaters were defined on the basis of consumption of red and processed meat >3 times or ≤3 times/wk. All characteristics are presented as age-adjusted means (5-y age groups) and as relative means, with regular meat eaters as the reference category. Estimates were modeled on the basis of linear regression. *For heel BMD, adjusted means were calculated on the basis of t-score, but relative means were plotted on the basis of the original values for interpretability. BMD, bone mineral density; bpm, beats per minute; DBP, diastolic blood pressure; SBP, systolic blood pressure.

**FIGURE 3:**
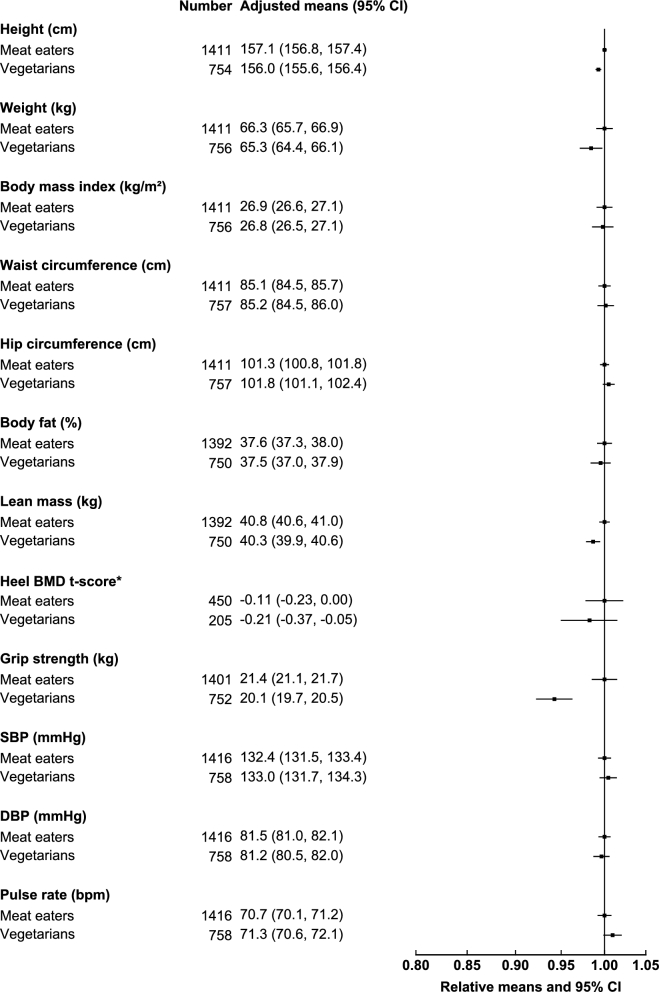
Anthropometric indexes and physiologic characteristics of British Indian women by diet group in the UK Biobank. All characteristics are presented as age-adjusted means (5-y age groups) and as relative means, with meat eaters as the reference category. Estimates were modeled on the basis of linear regression. *For heel BMD, adjusted means were calculated on the basis of t-score, but relative means were plotted on the basis of the original values for interpretability. BMD, bone mineral density; bpm, beats per minute; DBP, diastolic blood pressure; SBP, systolic blood pressure.

**FIGURE 4:**
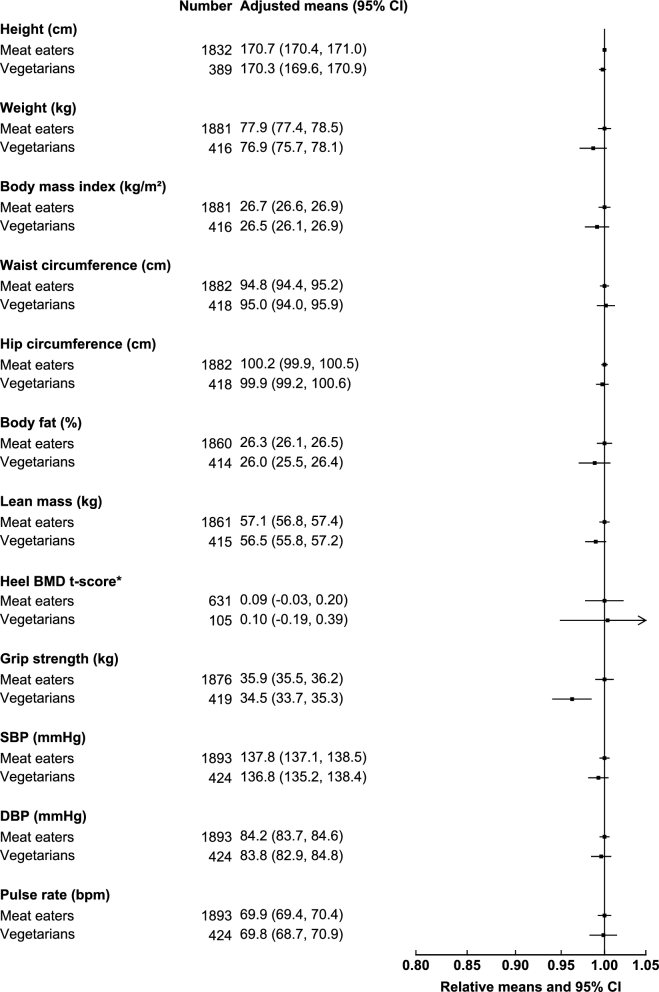
Anthropometric indexes and physiologic characteristics of British Indian men by diet group in the UK Biobank. All characteristics are presented as age-adjusted means (5-y age groups) and as relative means, with meat eaters as the reference category. Estimates were modeled on the basis of linear regression. *For heel BMD, adjusted means were calculated on the basis of t-score, but relative means were plotted on the basis of the original values for interpretability. BMD, bone mineral density; bpm, beats per minute; DBP, diastolic blood pressure; SBP, systolic blood pressure.

Anthropometric and physiologic characteristics of white and British Indian Biobank participants in the different diet groups are presented in **Figures 1****–****4**. For the white population, results reported in the following text represent significant differences after Bonferroni correction for multiple comparisons and by using regular meat eaters as the reference group (**Supplemental Tables 1 and 2**). Overall, in white women (Figure 1) and white men (Figure 2), low meat eaters, poultry eaters, fish eaters, vegetarians, and vegans had lower body weight (vegans compared with regular meat eaters: −7.2 kg in women and −9.1 kg in men), BMI (in kg/m^2^; −2.7 and −3.2), waist circumference (−4.8 and −7.3 cm), hip circumference (−5.3 and −4.4 cm), body fat percentage (−4.5% and −4.1%), and lean mass (−1.6 and −3.5 kg) compared with regular meat eaters. In women, poultry eaters, fish eaters, and vegetarians had a lower heel BMD t-score (−0.08 in vegetarians) than the regular meat eaters, and in men, low meat eaters had a higher heel BMD t-score (+0.05), but only the difference in poultry eaters in women remained significant upon adjustment for body weight.

For grip strength (higher value of either hand), compared with regular meat eaters white men who were low meat eaters had higher grip strength (+0.2 kg), whereas fish eaters and vegetarians had lower grip strength (−1.1 and −1.7 kg, respectively). The magnitudes of these differences were generally attenuated upon adjustment for height, lean mass, and physical activity, but remained significant in fish eaters and vegetarians. There was no significant difference in grip strength between regular meat eaters and the other diet groups in women. When we examined grip strength per kilogram of lean mass, the extreme diet groups (vegans and regular meat eaters) had similar values for both women and men. Results were similar when we examined average grip strength of the 2 hands instead of the higher value of either hand (results not shown).

All of the diet groups also had lower SBP (−4.2 and −6.1 mm Hg in white vegan women and men, respectively), and all non–red meat–eating groups had lower DBP (−3.3 and −4.5 mm Hg) compared with the regular meat eaters; these differences were attenuated but remained significant upon adjustment for percentage body fat. Compared with regular meat eaters, low meat eaters, poultry eaters, and fish eaters had a lower pulse rate, with fish eaters (−1.9 and −3.0 beats/min in women and men) having the lowest pulse rate overall.

In the British Indian participants, vegetarian women were shorter (−1.1 cm), had marginally lower body weight (−1.0 kg), and less lean mass (−0.5 kg) than Indian women who were meat eaters (Figure 3). Both British Indian vegetarian women and men (Figure 4) also had lower grip strength than meat eaters (−1.3 kg in women and −1.4 kg in men), and these differences were attenuated but remained significant upon adjustment for height, lean mass, and physical activity (**Supplemental Table 3**). Both vegetarian women (−0.03 kg grip strength/kg body weight) and men (−0.02 kg grip strength/kg body weight) had slightly lower grip strength per kilogram of lean mass than did meat eaters. No significant differences in the other anthropometric indexes and physiologic measures studied were observed in the British Indian populations.

## DISCUSSION

### Summary of results

In this large UK cohort, differences between diet groups in anthropometric and physiologic characteristics were observed in the white population but were less apparent in the British Indian population. Overall, white women and men who were poultry eaters, fish eaters, vegetarians, or vegans generally weighed less and had a lower BMI, waist and hip circumference, body fat percentage, and SBP and DBP than the regular meat eaters; and some non-meat eating diet groups had a lower heel BMD and pulse rate. Vegetarian men had lower overall grip strength but similar grip strength per kilogram of lean mass compared with meat eaters. British Indian vegetarian women were, on average, shorter and had a slightly lower body weight and lower lean mass than meat eaters, and both British Indian vegetarian women and men had lower grip strength than British Indian meat eaters.

### Comparison with other studies

Our findings were consistent with other studies that also reported lower BMI and waist circumference in vegetarians or vegans compared with meat eaters in white populations (1, 16–20), but the majority of previous studies compared only vegetarians with nonvegetarians without detailed categorization of the other diet groups. Of existing studies, only 1 study in the Adventist Health Study 2 compared BMI in 5 diet groups (nonvegetarians, semivegetarians, pesco-vegetarians, lacto-ovo-vegetarians, and vegans), and reported the lowest BMI in vegans (23.6), the highest BMI in nonvegetarians (28.8), and an intermediate BMI in the other diet groups, consistent with our results (20). In contrast, there is less agreement on the association between vegetarian diets and BMI in Indian populations, with 1 study reporting a higher BMI in Indian vegetarians (21.0) compared with Indian meat eaters (20.7) (21), and another study reporting the same BMI (23.9) in both diet groups (22). However, both of these studies examined Indians in India rather than British Indians, and the mean BMI in the first study was much lower than the average in the UK Biobank; therefore, our study population is not directly comparable.

Several studies have reported on differences in blood pressure associated with vegetarian diets, and a recent meta-analysis of 7 clinical trials and 32 observational studies reported that a vegetarian diet was associated with a reduction in mean SBP of 4.8 mm Hg (95% CI: 3.1–6.6 mm Hg) and in DBP of 2.2 mm Hg (95% CI: 1.0–3.5 mm Hg) on the basis of trial evidence and 6.9 mm Hg (95% CI: 4.7–9.1 mm Hg), and 4.7 mm Hg (95% CI: 3.1–6.3 mm Hg) on the basis of observational evidence, when compared with omnivorous diets (23). Of the studies included, the European Investigation into Cancer and Nutrition (EPIC)–Oxford study is another UK-based study with a large proportion of vegetarians, and in this study mean SBP and DBP were lowest in vegans and highest in meat eaters (age-adjusted difference of 2.6 and 4.2 mm Hg in SBP and 1.7 and 2.8 mm Hg in DBP for women and men, respectively) and intermediate in vegetarians and fish eaters, which is consistent with our results (2).

The associations of vegetarian diets with other anthropometric and physiologic characteristics are less well documented, and existing studies were small in size or results were inconclusive. For body composition, a small study in 105 Vietnamese Buddhist vegan nuns and 105 omnivorous women reported no significant difference in lean mass, fat mass, or percentage of fat mass between vegans and omnivores (24), in contrast to other small studies (total *n* < 100) from Asia, which reported lower body fat percentage in vegetarians (25, 26). For BMD, a meta-analysis of 9 studies in 2749 predominantly East Asian participants reported that lacto-ovo-vegetarian and vegan diets were associated with 2% and 6% lower BMD, respectively, at both the lumbar spine and femoral neck (4). For pulse rate, 1 study in 23 vegans and 24 omnivores reported faster daytime heart rate in vegans but no difference in sleep-time heart rate (5). No study was found that examined grip strength by habitual diet groups.

### Interpretation of findings and implications

In this large population cohort in the United Kingdom, there were substantial differences in various anthropometric and physiologic characteristics between different diet groups in white women and men, but few differences were apparent in British Indian women and men. The reason for this difference by ethnicity is not clear, although one possible explanation is that vegetarianism is a common dietary pattern among the Indian population and is predominantly driven by faith, cultural, and community reasons rather than health concerns (22). As a result, dietary choices may differ between white British and British Indian vegetarians, which subsequently result in differences in their health characteristics. In addition, British Indian meat eaters in the UK Biobank ate only small amounts of meat compared with the white meat eaters (Tables 1–3), which could also contribute to the smaller number of differences in anthropometric and physiologic characteristics between meat eaters and vegetarians in the Indian population. As another example of a distinct ethnic group, the Adventist Health Study 2 reported lower BMI and waist circumference in African American vegetarians than in nonvegetarians, although differences in blood pressure were not significant (27). Anthropometric and physiologic characteristics in vegetarians and nonvegetarians should be further studied in other nonwhite populations.

Differences in adiposity between diet groups may contribute to explaining the observed differences in blood pressure between the diet groups. In our analyses, the differences in SBP and DBP between diet groups were partially attenuated upon adjustment for body fat percentage, whereas in previous studies the same associations were partially attenuated upon adjustment for BMI (2). Previous studies found that higher sodium intake and lower potassium intake were associated with higher blood pressure (28, 29), but it is not clear whether there are differences in sodium or potassium intakes between vegetarian diets and diets that contain meat (30, 31). For pulse rate, it has been suggested that lower intakes of fish- or seafood-derived n–3 PUFAs may be associated with higher heart rate (5, 32), which would be consistent with the observed lower pulse rate in the poultry eaters and fish eaters in the UK Biobank, because these participants have the highest fish consumption.

It is well established that higher BMI and blood pressure increase the risk of cardiovascular diseases (7), and therefore we may expect non-meat eaters, who tend to have lower BMIs and blood pressure, to have a lower risk of this outcome. Evidence from both a pooled analysis and a UK study showed that vegetarians had a lower risk of ischemic heart disease (33, 34), and a US study showed that vegetarian men but not women had a lower risk of mortality from cardiovascular diseases than did nonvegetarians (35). Likewise, other anthropometric factors, such as higher percentage of body fat or, separately, higher pulse rate, have also been associated with higher cardiometabolic disease risk (36–38), and therefore the observed differences of these characteristics between the different diet groups may be important.

Although the associations of vegetarian diets with the other physiologic characteristics of interest have not been well studied, these characteristics are also important for long-term health. For example, BMD is a known predictor of fracture risk (8, 39, 40), and therefore lower BMD in some non-meat eaters may result in a higher fracture risk in these diet groups. Similarly, grip strength has been indicated to be a strong predictor of better health in relation to mortality, cardiovascular diseases, diabetes, and fracture risk (9, 10, 41, 42). In our study, differences in height, lean mass, and physical activity may have contributed to the differences in grip strength between diet groups, because the magnitudes of the differences were attenuated to some degree upon adjustment of these factors in both the white men and the British Indian population, and because vegans in the white population had a lower overall grip strength but a similar grip strength per kilogram of lean mass.

### Strengths and limitations

The strength of this study is that it included a large sample size of close to 500,000 white and 5000 British Indian participants in the United Kingdom and reported on a range of anthropometric indexes and physiologic characteristics that were objectively measured following standard protocols, thereby minimizing the chance of reporting bias. In the white population, categorization of diet was performed in 6 groups, which allowed detailed analyses of characteristics in persons across a range of dietary habits. Despite the large overall sample size, the numbers of white vegans and British Indian vegetarians were relatively small, and therefore the possibility of false-negative findings cannot be ruled out. Because diet group categorization was based on data collected at 1 time point, and did not take into account the length of time the participants have been in any particular diet group, misclassification bias is possible, although reported long-term abstinence from meat in this cohort was high (92% of non-meat eaters overall with >5 y self-reported adherence to not eating meat). Measurement error related to the outcomes was also possible, and little is known of the validity of bioimpedance measures of extremely underweight (BMI <14) or obese (BMI ≥36) participants (5% of total cohort) (43). As with all observational studies, some degree of self-selection bias may be present (13), and there was no information to indicate whether vegetarians might be more likely to respond. Because the analyses were based on diets of participants in one country, the results may not be generalizable to other populations or cultures, as indicated by differences between white British and British Indian vegetarians in our study. Because the study is cross-sectional, it was not possible to determine causality, and residual confounding by other dietary or nondietary factors may be present.

### Conclusions

In this large-population cohort in the United Kingdom, white women and men who were poultry eaters, fish eaters, vegetarians, or vegans generally had lower adiposity, BMD, grip strength, blood pressure, and pulse rate than did white women and men who were regular meat eaters. However, there were fewer differences in anthropometric and physiologic characteristics in British Indian meat eaters and vegetarians, perhaps due to the greater similarities between the 2 diets in this population. Because the characteristics included in this study are known risk factors of long-term disease risk, the observed differences in these characteristics between diet groups may be important in determining long-term health in individuals of different dietary habits.

## Supplementary Material

Supplementary Data

## Notes

Supported by the UK Medical Research Council (MR/M012190/1) and Wellcome Trust Our Planet Our Health [Livestock, Environment and People (LEAP) 205212/Z/16/Z]. KEB is supported by the Girdlers’ New Zealand Health Research Council Fellowship. JGS was supported by private sponsorship covering living expenses from Andrzej and Beata Gajek.

This research was conducted using the UK Biobank Resource under application 3037. The UK Biobank is an open-access resource. Bona fide researchers can apply to use the UK Biobank data set by registering and applying at http://ukbiobank.ac.uk/register-apply/.

Supplemental Tables 1–3 and Supplemental Figure 1 are available from the “Supplementary data” link in the online posting of the article and from the same link in the online table of contents at https://academic.oup.com/ajcn/.

Abbreviations used: BMD, bone mineral density; DBP, diastolic blood pressure; SBP, systolic blood pressure.
