# Supplementary material for: Anthropometric and physiologic characteristics in white and British Indian vegetarians and nonvegetarians in the UK Biobank
Source: Am J Clin Nutr. 2018 Jun 4;107(6):909–20. doi: 10.1093/ajcn/nqy042 (PMC5985736; doi:10.1093/ajcn/nqy042)
Supplement: Supplementary Data [file nqy042_supp.docx]

502,619 participants provided informed consent

Excluded 57,907 participants if they reported changing diet in the past 5 years due to illness, or did not answer the question

444,712 participants

Excluded 18,547 participants of other ethnicities

421,379 white participants and 4,786 Indian participants

Excluded 2,630 white participants and 278 Indian participants who could not be classified into a diet group

418,749 white participants and 4,508 Indian participants

Supplemental figure 1: Participant flow chart of the study.

Supplemental table 1. Anthropometric indices and physiological characteristics of white women by diet group in the UK Biobank, with adjustment for potential confounders (n=229,806).

| **Characteristics** | **Meat eaters** | | | **Fish eaters**  **Max N=6,988** | **Vegetarians**  **Max N=4,305** | **Vegans**  **Max N=223** |
| --- | --- | --- | --- | --- | --- | --- |
|  | **Regular consumption**  **(>3 times/week)*^1^***  **Max N=86,432** | **Low consumption**  **(≤3 times/week)*^1^***  **Max N=128,429** | **Poultry eaters**  **Max N=3,429** |  |  |  |
| **Height (cm)** | 162.6 (162.6, 162.6)^a^ | 162.8 (162.7, 162.8)^b^ | 162.7 (162.5, 162.9)^ab^ | 163.5 (163.3, 163.6)^c^ | 163.0 (162.8, 163.2)^b^ | 162.4 (161.6, 163.2)^abc^ |
| **Weight (kg)** | 72.6 (72.5, 72.7)^c^ | 70.4 (70.4, 70.5)^b^ | 66.3 (65.9, 66.8)^a^ | 66.9 (66.5, 67.2)^a^ | 67.1 (66.7, 67.5)^a^ | 65.4 (63.6, 67.1)^a^ |
| **Body mass index (kg/m^2^)** | 27.5 (27.4, 27.5)^c^ | 26.6 (26.6, 26.6)^b^ | 25.1 (24.9, 25.2)^a^ | 25.0 (24.9, 25.2)^a^ | 25.3 (25.1, 25.4)^a^ | 24.8 (24.2, 25.5)^a^ |
| **Waist circumference (cm)** | 85.6 (85.6, 85.7)^d^ | 83.4 (83.3, 83.5)^c^ | 79.5 (79.1, 79.9)^a^ | 80.1 (79.8, 80.4)^a^ | 80.9 (80.5, 81.3)^b^ | 80.8 (79.2, 82.4)^ab^ |
| **Hip circumference (cm)** | 104.2 (104.1, 104.3)^d^ | 102.6 (102.6, 102.7)^c^ | 99.5 (99.2, 99.9)^a^ | 100.1 (99.9, 100.3)^ab^ | 100.5 (100.2, 100.8)^b^ | 98.9 (97.6, 100.3)^ab^ |
| **Body fat (%)** | 37.2 (37.1, 37.2)^d^ | 36.0 (36.0, 36.1)^c^ | 33.4 (33.2, 33.7)^a^ | 33.5 (33.4, 33.7)^a^ | 34.0 (33.8, 34.2)^b^ | 32.7 (31.8, 33.6)^ab^ |
| **Lean mass (kg)** | 44.8 (44.8, 44.9)^d^ | 44.3 (44.3, 44.4)^c^ | 43.4 (43.3, 43.6)^a^ | 43.8 (43.6, 43.9)^b^ | 43.5 (43.4, 43.7)^ab^ | 43.2 (42.5, 43.8)^ab^ |
| **Heel bone mineral density** | 0.52 (0.52, 0.52)^b^ | 0.52 (0.52, 0.52)^b^ | 0.51 (0.50, 0.51)^a^ | 0.51 (0.51, 0.51)^a^ | 0.51 (0.50, 0.51)^a^ | 0.49 (0.47, 0.51)^ab^ |
| **Heel bone mineral density t-score** | -0.54 (-0.55, -0.53)^b^ | -0.55 (-0.56, -0.55)^b^ | -0.65 (-0.69, -0.61)^a^ | -0.62 (-0.65, -0.59)^a^ | -0.62 (-0.66, -0.58)^a^ | -0.80 (-0.98, -0.62)^ab^ |
| *+* weight | -0.55 (-0.55, -0.54)^b^ | -0.55 (-0.56, -0.55)^b^ | -0.63 (-0.67, -0.59)^a^ | -0.60 (-0.63, -0.57)^ab^ | -0.61 (-0.65, -0.57)^ab^ | -0.77 (-0.95, -0.59)^ab^ |
| **Grip strength (kg)*^2^*** | 25.3 (25.3, 25.3)^ab^ | 25.4 (25.4, 25.4)^b^ | 25.1 (24.9, 25.3)^ab^ | 25.5 (25.3, 25.6)^b^ | 25.1 (24.9, 25.3)^a^ | 24.5 (23.7, 25.3)^ab^ |
| *+* height | 25.3 (25.3, 25.4)^b^ | 25.4 (25.4, 25.4)^b^ | 25.1 (25.0, 25.3)^ab^ | 25.3 (25.1, 25.4)^ab^ | 25.0 (24.9, 25.2)^a^ | 24.6 (23.8, 25.3)^ab^ |
| *+* lean mass | 25.3 (25.2, 25.3)^a^ | 25.4 (25.4, 25.4)^a^ | 25.3 (25.1, 25.5)^a^ | 25.4 (25.3, 25.6)^a^ | 25.2 (25.0, 25.4)^a^ | 24.8 (24.1, 25.5)^a^ |
| *+* physical activity | 25.3 (25.3, 25.3)^a^ | 25.4 (25.4, 25.4)^a^ | 25.2 (25.0, 25.4)^a^ | 25.4 (25.2, 25.5)^a^ | 25.2 (25.0, 25.3)^a^ | 24.7 (24.0, 25.4)^a^ |
| **Grip strength/lean mass (kg/kg)** | 0.57 (0.57, 0.57)^a^ | 0.58 (0.57, 0.58)^b^ | 0.58 (0.58, 0.59)^bc^ | 0.58 (0.58, 0.59)^c^ | 0.58 (0.58, 0.58)^bc^ | 0.56 (0.55, 0.58)^abc^ |
| **Systolic blood pressure (mmHg)** | 136.2 (136.1, 136.3)^c^ | 135.0 (134.9, 135.1)^b^ | 132.8 (132.2, 133.4)^a^ | 132.7 (132.2, 133.1)^a^ | 133.3 (132.8, 133.8)^a^ | 132.0 (129.7, 134.3)^ab^ |
| *+* body fat % | 135.8 (135.7, 135.9)^c^ | 135.1 (135.0, 135.2)^b^ | 134.0 (133.4, 134.6)^a^ | 133.9 (133.4, 134.3)^a^ | 134.3 (133.7, 134.8)^a^ | 133.4 (131.1, 135.7)^abc^ |
| **Diastolic blood pressure (mmHg)** | 81.1 (81.1, 81.2)^d^ | 80.5 (80.4, 80.5)^c^ | 78.9 (78.5, 79.2)^a^ | 79.1 (78.8, 79.3)^ab^ | 79.6 (79.3, 79.9)^b^ | 77.8 (76.5, 79.1)^ab^ |
| *+* body fat % | 80.8 (80.7, 80.8)^b^ | 80.6 (80.5, 80.6)^b^ | 80.0 (79.6, 80.3)^a^ | 80.2 (79.9, 80.4)^a^ | 80.5 (80.2, 80.8)^ab^ | 79.0 (77.8, 80.3)^ab^ |
| **Pulse rate (bpm)** | 70.5 (70.4, 70.6)^c^ | 69.8 (69.8, 69.9)^b^ | 68.9 (68.6, 69.3)^a^ | 68.6 (68.3, 68.8)^a^ | 70.1 (69.8, 70.4)^bc^ | 70.6 (69.2, 71.9)^abc^ |
| *+* body fat % | 70.3 (70.2, 70.4)^c^ | 69.9 (69.9, 70.0)^b^ | 69.5 (69.1, 69.8)^ab^ | 69.1 (68.9, 69.4)^a^ | 70.5 (70.2, 70.9)^c^ | 71.1 (69.8, 72.5)^abc^ |

All estimates were expressed as adjusted means (95% confidence intervals) with adjustment for age (5 year age groups), and additionally for variable specified in row. Lean mass, Height and body weight were adjusted for categorically as 2.5 unit increments; physical activity was adjusted for categorically as 5 unit increments in excess MET-hours per week. Groups that do not share a superscript letter were significantly different at the 5% level from post hoc pairwise comparisons based on linear regression models, and after Bonferroni correction for multiple comparisons.
1. Includes participants who consume any red or processed meat, regardless of whether they consume poultry, fish, or dairy. Cut-offs of regular and low consumption determined based on consumption of red and processed meat (beef, lamb, pork, processed meat) as reported on the touchscreen questionnaire.
2. Higher grip strength value of either hand.

Supplemental table 2. Anthropometric indices and physiological characteristics of white men by diet group in the UK Biobank, with adjustment for potential confounders (n=188,934).

| **Characteristics** | **Meat eaters** | | | **Fish eaters**  **Max N=2,686** | **Vegetarians**  **Max N=2,061** | **Vegans**  **Max N=155** |
| --- | --- | --- | --- | --- | --- | --- |
|  | **Regular consumption**  **(>3 times/week)*^1^***  **Max N=111,734** | **Low consumption**  **(≤3 times/week)*^1^***  **Max N=71,355** | **Poultry eaters**  **Max N=952** |  |  |  |
| **Height (cm)** | 176.0 (175.9, 176.0)^a^ | 176.1 (176.0, 176.1)^b^ | 176.2 (175.8, 176.6)^ab^ | 176.4 (176.2, 176.7)^b^ | 176.3 (176.0, 176.6)^ab^ | 176.6 (175.6, 177.7)^ab^ |
| **Weight (kg)** | 86.7 (86.6, 86.8)^c^ | 84.8 (84.7, 84.9)^b^ | 80.6 (79.7, 81.5)^a^ | 80.0 (79.5, 80.5)^a^ | 80.2 (79.6, 80.8)^a^ | 77.6 (75.4, 79.7)^a^ |
| **Body mass index (kg/m^2^)** | 28.0 (28.0, 28.0)^d^ | 27.3 (27.3, 27.4)^c^ | 26.0 (25.7, 26.2)^b^ | 25.7 (25.6, 25.9)^ab^ | 25.8 (25.6, 26.0)^ab^ | 24.8 (24.2, 25.5)^a^ |
| **Waist circumference (cm)** | 97.5 (97.5, 97.6)^c^ | 95.4 (95.3, 95.5)^b^ | 91.4 (90.7, 92.1)^a^ | 91.5 (91.1, 91.9)^a^ | 92.3 (91.8, 92.8)^a^ | 90.2 (88.5, 92.0)^a^ |
| **Hip circumference (cm)** | 103.8 (103.7, 103.8)^c^ | 102.9 (102.8, 102.9)^b^ | 100.7 (100.2, 101.2)^a^ | 100.6 (100.3, 100.8)^a^ | 100.8 (100.5, 101.1)^a^ | 99.4 (98.2, 100.6)^a^ |
| **Body fat (%)** | 25.4 (25.4, 25.5)^d^ | 24.5 (24.5, 24.6)^c^ | 22.5 (22.1, 22.8)^ab^ | 22.4 (22.2, 22.6)^a^ | 22.9 (22.7, 23.1)^b^ | 21.3 (20.4, 22.2)^a^ |
| **Lean mass (kg)** | 64.1 (64.0, 64.1)^c^ | 63.5 (63.4, 63.6)^b^ | 62.0 (61.6, 62.5)^a^ | 61.7 (61.4, 62.0)^a^ | 61.3 (61.0, 61.7)^a^ | 60.6 (59.5, 61.8)^a^ |
| **Heel bone mineral density** | 0.57 (0.57, 0.57)^a^ | 0.58 (0.57, 0.58)^b^ | 0.56 (0.55, 0.57)^ab^ | 0.56 (0.56, 0.57)^a^ | 0.56 (0.55, 0.57)^a^ | 0.53 (0.50, 0.56)^a^ |
| **Heel bone mineral density t-score** | -0.09 (-0.09, -0.08)^a^ | -0.04 (-0.06, -0.03)^b^ | -0.18 (-0.29, -0.08)^ab^ | -0.16 (-0.22, -0.10)^a^ | -0.18 (-0.25, -0.11)^a^ | -0.48 (-0.75, -0.21)^a^ |
| *+* weight | -0.09 (-0.10, -0.08)^a^ | -0.04 (-0.05, -0.03)^a^ | -0.14 (-0.24, -0.03)^a^ | -0.11 (-0.17, -0.05)^a^ | -0.13 (-0.20, -0.06)^a^ | -0.37 (-0.64, -0.10)^a^ |
| **Grip strength (kg)*^2^*** | 42.2 (42.1, 42.2)^c^ | 42.4 (42.3, 42.4)^d^ | 41.5 (40.9, 42.0)^bc^ | 41.1 (40.8, 41.5)^ab^ | 40.5 (40.1, 40.8)^a^ | 40.3 (39.0, 41.7)^abc^ |
| *+* height | 42.2 (42.1, 42.2)^d^ | 42.3 (42.3, 42.4)^c^ | 41.4 (40.9, 41.9)^b^ | 41.0 (40.7, 41.3)^ab^ | 40.4 (40.0, 40.7)^a^ | 40.2 (38.9, 41.5)^ab^ |
| *+* lean mass | 42.1 (42.1, 42.2)^b^ | 42.4 (42.3, 42.4)^b^ | 41.8 (41.3, 42.3)^ab^ | 41.5 (41.2, 41.8)^a^ | 41.0 (40.6, 41.3)^a^ | 41.0 (39.7, 42.2)^ab^ |
| *+* physical activity | 42.1 (42.1, 42.2)^b^ | 42.4 (42.3, 42.4)^b^ | 41.7 (41.1, 42.2)^ab^ | 41.4 (41.1, 41.7)^a^ | 40.9 (40.6, 41.3)^a^ | 40.8 (39.6, 42.1)^ab^ |
| **Grip strength/lean mass (kg/kg)** | 0.66 (0.66, 0.66)^a^ | 0.67 (0.67, 0.67)^b^ | 0.67 (0.66, 0.68)^ab^ | 0.67 (0.66, 0.67)^b^ | 0.66 (0.66, 0.67)^ab^ | 0.67 (0.65, 0.69)^ab^ |
| **Systolic blood pressure (mmHg)** | 141.7 (141.6, 141.8)^c^ | 140.8 (140.7, 141.0)^b^ | 139.3 (138.2, 140.3)^ab^ | 138.3 (137.7, 138.9)^a^ | 139.1 (138.3, 139.8)^a^ | 135.6 (132.9, 138.2)^a^ |
| *+* body fat % | 141.5 (141.4, 141.6)^c^ | 141.0 (140.9, 141.1)^b^ | 140.5 (139.5, 141.6)^abc^ | 139.6 (139.0, 140.2)^a^ | 140.1 (139.4, 140.8)^ab^ | 137.4 (134.8, 140.0)^ab^ |
| **Diastolic blood pressure (mmHg)** | 84.6 (84.6, 84.7)^d^ | 84.0 (83.9, 84.1)^c^ | 82.5 (81.9, 83.1)^ab^ | 82.4 (82.0, 82.8)^ab^ | 83.0 (82.6, 83.5)^b^ | 80.1 (78.6, 81.7)^a^ |
| *+* body fat % | 84.4 (84.4, 84.5)^b^ | 84.2 (84.1, 84.3)^b^ | 83.7 (83.1, 84.3)^ab^ | 83.6 (83.3, 84.0)^a^ | 84.0 (83.6, 84.4)^ab^ | 81.8 (80.3, 83.3)^a^ |
| **Pulse rate (bpm)** | 68.7 (68.6, 68.7)^d^ | 67.3 (67.2, 67.4)^bc^ | 66.4 (65.7, 67.2)^ab^ | 65.7 (65.3, 66.2)^a^ | 67.9 (67.4, 68.4)^cd^ | 66.4 (64.6, 68.3)^abcd^ |
| *+* body fat % | 68.5 (68.4, 68.6)^c^ | 67.5 (67.4, 67.6)^b^ | 67.3 (66.5, 68.0)^ab^ | 66.6 (66.2, 67.1)^a^ | 68.6 (68.1, 69.1)^c^ | 67.6 (65.8, 69.4)^abc^ |

All estimates were expressed as adjusted means (95% confidence intervals) with adjustment for age (5 year age groups), and additionally for variable specified in row. Lean mass, Height and body weight were adjusted for categorically as 2.5 unit increments; physical activity was adjusted for categorically as 5 unit increments in excess MET-hours per week. Groups that do not share a superscript letter were significantly different at the 5% level from post hoc pairwise comparisons based on linear regression models, and after Bonferroni correction for multiple comparisons.
1. Includes participants who consume any red or processed meat, regardless of whether they consume poultry, fish, or dairy. Cut-offs of regular and low consumption determined based on consumption of red and processed meat (beef, lamb, pork, processed meat) as reported on the touchscreen questionnaire.
2. Higher grip strength value of either hand.

Supplemental table 3. Anthropometric indices and physiological characteristics of British Indian women and men by diet group in the UK Biobank, with adjustment for potential confounders (n women=2,183, n men=2,325).

| **Characteristics** | **Women** | | **Men** | |
| --- | --- | --- | --- | --- |
|  | **Meat eaters**  **Max N=1,422** | **Vegetarians**  **Max N=761** | **Meat eaters**  **Max N=1,900** | **Vegetarians**  **Max N=425** |
| **Height (cm)** | 157.1 (156.8, 157.4)^b^ | 156.0 (155.6, 156.4)^a^ | 170.7 (170.4, 171.0)^a^ | 170.3 (169.6, 170.9)^a^ |
| **Weight (kg)** | 66.3 (65.7, 66.9)^b^ | 65.3 (64.4, 66.1)^a^ | 77.9 (77.4, 78.5)^a^ | 76.9 (75.7, 78.1)^a^ |
| **Body mass index (kg/m^2^)** | 26.9 (26.6, 27.1)^a^ | 26.8 (26.5, 27.1)^a^ | 26.7 (26.6, 26.9)^a^ | 26.5 (26.1, 26.9)^a^ |
| **Waist circumference (cm)** | 85.1 (84.5, 85.7)^a^ | 85.2 (84.5, 86.0)^a^ | 94.8 (94.4, 95.2)^a^ | 95.0 (94.0, 95.9)^a^ |
| **Hip circumference (cm)** | 101.3 (100.8, 101.8)^a^ | 101.8 (101.1, 102.4)^a^ | 100.2 (99.9, 100.5)^a^ | 99.9 (99.2, 100.6)^a^ |
| **Body fat (%)** | 37.6 (37.3, 38.0)^a^ | 37.5 (37.0, 37.9)^a^ | 26.3 (26.1, 26.5)^a^ | 26.0 (25.5, 26.4)^a^ |
| **Lean mass (kg)** | 40.8 (40.6, 41.0)^b^ | 40.3 (39.9, 40.6)^a^ | 57.1 (56.8, 57.4)^a^ | 56.5 (55.8, 57.2)^a^ |
| **Heel bone mineral density** | 0.57 (0.55, 0.58)^a^ | 0.56 (0.54, 0.57)^a^ | 0.59 (0.58, 0.60)^a^ | 0.59 (0.56, 0.63)^a^ |
| **Heel bone mineral density t-score** | -0.11 (-0.23, 0.00)^a^ | -0.21 (-0.37, -0.05)^a^ | 0.09 (-0.03, 0.20)^a^ | 0.10 (-0.19, 0.39)^a^ |
| *+* weight | -0.11 (-0.22, -0.01)^a^ | -0.21 (-0.37, -0.05)^a^ | 0.09 (-0.03, 0.20)^a^ | 0.10 (-0.19, 0.38)^a^ |
| **Grip strength (kg)*^1^*** | 21.4 (21.1, 21.7)^b^ | 20.1 (19.7, 20.5)^a^ | 35.9 (35.5, 36.2)^b^ | 34.5 (33.7, 35.3)^a^ |
| *+* height | 21.2 (20.9, 21.5)^b^ | 20.3 (19.9, 20.7)^a^ | 35.8 (35.5, 36.2)^b^ | 34.7 (33.9, 35.5)^a^ |
| *+* lean mass | 21.2 (20.9, 21.5)^b^ | 20.4 (19.9, 20.8)^a^ | 35.8 (35.5, 36.2)^b^ | 34.8 (34.0, 35.5)^a^ |
| *+* physical activity | 21.2 (20.9, 21.5)^b^ | 20.4 (20.0, 20.8)^a^ | 35.8 (35.5, 36.2)^b^ | 34.8 (34.0, 35.5)^a^ |
| **Grip strength/lean mass (kg/kg)** | 0.53 (0.52, 0.53)^b^ | 0.50 (0.49, 0.51)^a^ | 0.63 (0.62, 0.64)^b^ | 0.61 (0.60, 0.63)^a^ |
| **Systolic blood pressure (mmHg)** | 132.4 (131.5, 133.4)^a^ | 133.0 (131.7, 134.3)^a^ | 137.8 (137.1, 138.5)^a^ | 136.8 (135.2, 138.4)^a^ |
| *+* body fat % | 132.4 (131.5, 133.3)^a^ | 133.1 (131.8, 134.3)^a^ | 137.8 (137.0, 138.5)^a^ | 137.0 (135.4, 138.5)^a^ |
| **Diastolic blood pressure**  **(mmHg)** | 81.5 (81.0, 82.1)^a^ | 81.2 (80.5, 82.0)^a^ | 84.2 (83.7, 84.6)^a^ | 83.8 (82.9, 84.8)^a^ |
| *+* body fat % | 81.5 (81.0, 82.0)^a^ | 81.3 (80.5, 82.0)^a^ | 84.1 (83.7, 84.6)^a^ | 84.0 (83.0, 84.9)^a^ |
| **Pulse rate (bpm)** | 70.7 (70.1, 71.2)^a^ | 71.3 (70.6, 72.1)^a^ | 69.9 (69.4, 70.4)^a^ | 69.8 (68.7, 70.9)^a^ |
| *+* body fat % | 70.7 (70.1, 71.2)^a^ | 71.3 (70.6, 72.1)^a^ | 69.9 (69.4, 70.4)^a^ | 69.9 (68.8, 71.0)^a^ |

All estimates were expressed as adjusted means (95% confidence intervals) with adjustment for age (5 year age groups), and additionally for variable specified in row. Lean mass, Height and body weight were adjusted for categorically as 2.5 unit increments; physical activity was adjusted for categorically as 5 unit increments in excess MET-hours per week. Groups that do not share a superscript letter were significantly different at the 5% level from post hoc pairwise comparisons based on linear regression models. Comparisons were made separately for women and men.
1. Higher grip strength value of either hand.
